# Supplementary material for: Adenovirus-mediated expression of SIK1 improves hepatic glucose and lipid metabolism in type 2 diabetes mellitus rats
Source: PLoS One. 2019 Jun 24;14(6):e0210930. doi: 10.1371/journal.pone.0210930 (PMC6590778; doi:10.1371/journal.pone.0210930)
Supplement: S1 Appendix — (DOCX) [file pone.0210930.s001.docx]

**Supplementary Methods**

Recombinant Adenovirus Constructs

SIK1 cDNA (NM_021693) was obtained from the cDNA library of Genechem (Shanghai, China). The 2,337 base pair PCR product was cloned into a linearised adenovirus plasmid GV314 (Genechem) with T4 DNA ligase and transfected into competent Escherichia coli cells. Positive clones were selected by ampicillin resistance and then sequenced by ABI3730 sequencing analysis (Invitrogen, Shanghai, China). The SIK1 overexpression adenovirus (Ad-SIK1) was packaged in HEK293T cells and purified with an Adeno-X Virus Purification Kit (BD Biosciences, San Jose, CA, USA). The endpoint dilution method was used to determine the viral titre. Adenovirus particles containing CMV-MCS-3FLAG-SV40-EGFP (Ad-GFP; purchased from Genechem) served as a negative control.
